# Supplementary material for: The Effect of Aspirin Use on Incident Hepatocellular Carcinoma—An Updated Systematic Review and Meta-Analysis
Source: Cancers (Basel). 2023 Jul 6;15(13):3518. doi: 10.3390/cancers15133518 (PMC10341252; doi:10.3390/cancers15133518)
Supplement: Supplementary file 1 [file cancers-15-03518-s001.zip › cancers-2464685-supplementary.pdf]

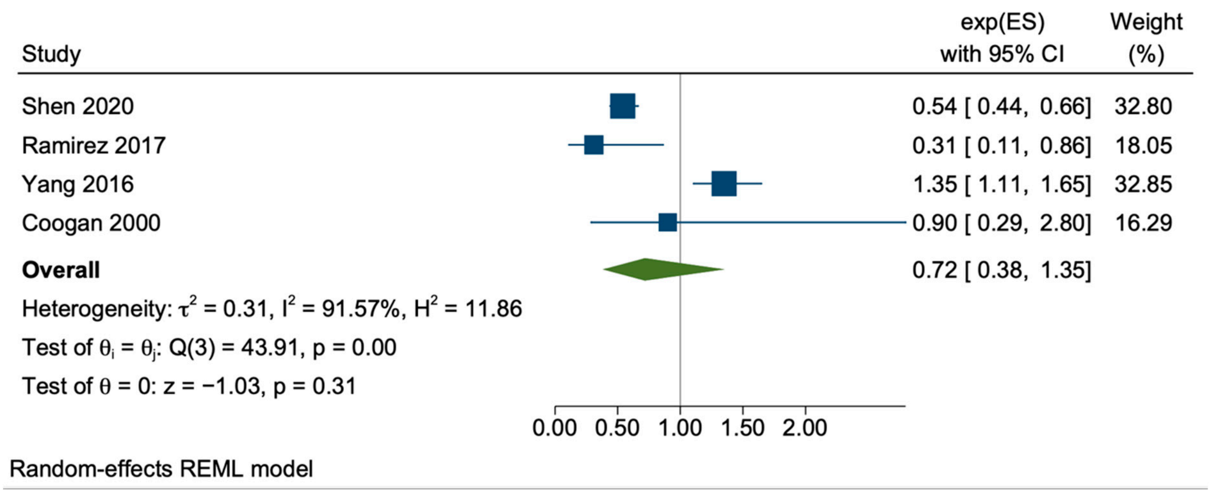

Figure S1. Random-effects meta-analysis of the pooled case-control studies, utilising only crude unadjusted data [46–49].

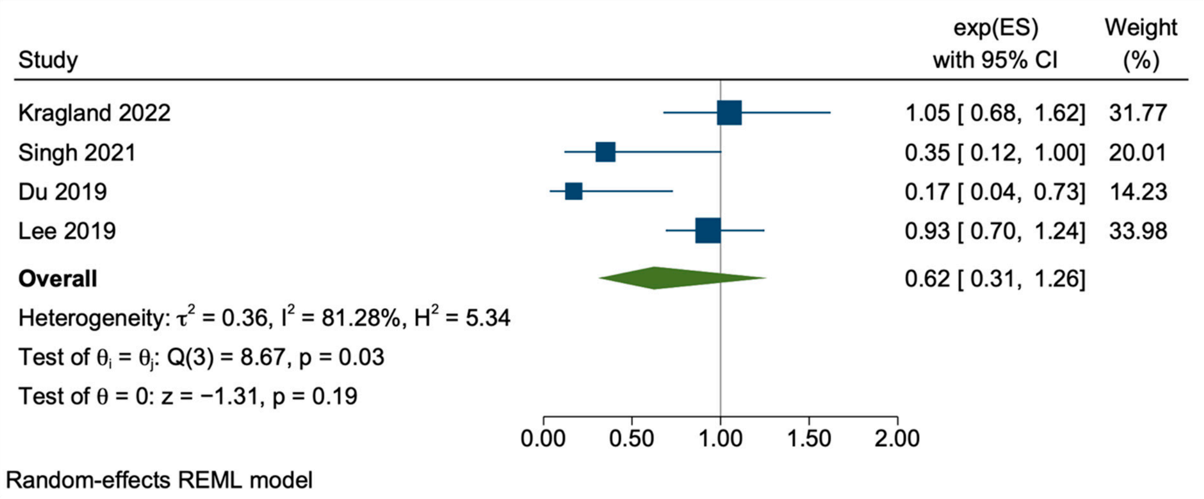

Figure S2. Subgroup random-effects meta-analysis of patients with cirrhosis, utilising only crude unadjusted data [34,37–39,44].
